# Antigenic divergence of cobra short-chain α-neurotoxins: Implications for regional antivenom effectiveness in Southeast Asia

**DOI:** 10.1371/journal.pntd.0013859

**Published:** 2026-05-14

**Authors:** Choo Hock Tan, Praneetha Palasuberniam, Lee Louisa Pernee, Kae Yi Tan

**Affiliations:** 1 School of Medicine, College of Life Sciences and Medicine, National Tsing Hua University, Hsinchu, Taiwan; 2 Institute of Bioinformatics and Structural Biology, College of Life Sciences and Medicine, National Tsing Hua University, Hsinchu, Taiwan; 3 Department of Medical Sciences, College of Life Sciences and Medicine, National Tsing Hua University, Hsinchu, Taiwan; 4 Venom Research & Toxicology Research Lab, Department of Pharmacology, Faculty of Medicine, University of Malaya, Kuala Lumpur, Malaysia; 5 Department of Biomedical Sciences, Faculty of Medicine and Health Sciences, Universiti Malaysia Sabah, Kota Kinabalu, Sabah, Malaysia; 6 School of Biological and Chemical Engineering, NingboTech University, Ningbo, Zhejiang, China; 7 Protein and Interactomics Lab, Department of Molecular Medicine, Faculty of Medicine, University of Malaya, Kuala Lumpur, Malaysia; Fundação de Medicina Tropical Doutor Heitor Vieira Dourado: Fundacao de Medicina Tropical Doutor Heitor Vieira Dourado, BRAZIL

## Abstract

Short-chain α-neurotoxins (SNTXs) are the principal neurotoxic components in several Asiatic cobras, including *Naja atra*, *Naja philippinensis*, and *Naja samarensis*. Although structurally conserved, SNTXs exhibit marked antigenic variation that can limit the effectiveness of regional antivenoms used for snakebite envenoming in Asia. Here, we evaluated the immunoreactivity of the Philippine Cobra Antivenom (PCAV) and three regional products—*Naja kaouthia* Monovalent Antivenom (NkMAV), Neuro Bivalent Antivenom (NBAV), and Indonesian SABU—against a panel of cobra venoms and purified α-neurotoxins. PCAV bound strongly to homologous *N. philippinensis* SNTX but showed significantly lower cross-reactivity with SNTXs from *Naja kaouthia*, *Naja sputatrix*, and *N. atra*, and minimal recognition of marine elapid SNTXs and long-chain α-neurotoxins (LNTXs) of Monocled Cobra as well as King Cobra. Conversely, NkMAV and NBAV recognized mainland Asian SNTXs more broadly but reacted poorly with the Philippine cobra toxin. These differences were statistically significant in multiple comparisons *versus* the homologous venom control. Hierarchical clustering of normalized immunoreactivity delineated two major SNTX antigenic subtypes corresponding to Philippine *versus* continental Asian lineages. Peptide sequence analysis unmasked two distinct loop-II motifs (^28^WWS–TII^37^ and ^28^RWR–YRT^37^) associated with these divergent immunotypes. Phylogenetic reconstruction suggests that the Philippine cobras retain an ancestral-like SNTX motif, while Sundaic and East Asian cobras have diversified to employ another major SNTX form. Epitope prediction further revealed differences in surface exposure and accessibility, which helps explain the limited cross-neutralization among antivenoms. These findings demonstrate a clear antigenic dichotomy among Asian cobra SNTXs, which underlies species-specific antivenom effectiveness and highlights the need to incorporate representative SNTX variants into immunogen formulations to improve regional antivenom coverage.

## Author summary

Snakebite envenoming by Asiatic cobras is a major public health problem, where paralysis is primarily caused by short-chain α-neurotoxins (SNTXs). Antivenoms are the only specific treatment, but their effectiveness often varies across regions. In this study, we examined how well different Asian antivenoms recognize SNTXs from several cobra species. We found that antivenoms tend to work best against the species they were raised against, but show reduced effectiveness against toxins from other regions. Notably, the Philippine Cobra Antivenom strongly recognized toxins from the Philippine cobra but poorly detected those from mainland Asian cobras, while mainland antivenoms showed the opposite pattern. By analyzing toxin sequences, we identified two distinct molecular signatures in a critical toxin region (loop II), which define separate antigenic groups corresponding to Philippine and continental Asian cobras. These differences influence how accessible the toxin surfaces are to antibodies, helping to explain why cross-neutralization across different species and regions is limited. Together, our findings reveal a clear antigenic divide among cobra neurotoxins in Asia, which directly impacts antivenom performance. This highlights the importance of incorporating diverse toxin variants when producing antivenom, so that treaments can provide broader and more effective protection across regions.

## Introduction

Snakebite envenoming is a WHO-listed priority neglected tropical disease that disproportionately affects rural and low-income communities across Asia [1]. As a One Health challenge spanning human health, biodiversity, and regional healthcare inequities, snakebite envenoming requires improved antivenom coverage informed by a deeper understanding of venom variation among medically important snakes [2,3]. Cobra venoms, in particular, exhibit striking interspecific and geographical diversity in toxin composition, which directly influences clinical severity and treatment outcomes [3]. Within these venoms, three-fingered protein toxins represent the dominant neurotoxic components responsible for rapid paralysis in neurotoxic snakebites. Proteins with the three-finger fold are widely distributed among metazoans and play critical physiological roles. For instance, the GPI-anchored Lynx1 protein modulates cholinergic transmission by fine-tuning nicotinic acetylcholine receptors (nAChRs) in mammals, influencing learning, memory, and neural plasticity. Similarly, secretory three-finger proteins (TFPs) such as SLURP-1 and SLURP-2 are involved in cell proliferation, immune responses, and tissue repair [4–6]. In some advanced snakes, these ancestral physiological proteins were co-opted for neofunctionalization, as the inherent plasticity of the three-finger motif allowed it to be exploited and repurposed for diverse toxic effects, including cytotoxicity to neurotoxicity [7,8]. This evolutionary adaptation has given rise to a new class of TFPs known as three-finger toxins (3FTXs), which are predominantly found in the Elapidae family, including cobras, kraits, king cobras, coral snakes, mambas, and sea snakes [3–9,11].

Within the 3FTX family, alpha-neurotoxins are among the most well-studied members. These toxins act as high-affinity competitive blockers of postsynaptic muscle-type nAChRs [12]. The alpha-neurotoxins are broadly classified into two subfamilies based on peptide length and the number of intramolecular disulfide bonds: (1) short-chain α-neurotoxins (SNTX), which have 60–62 residues and four disulfide bridges; and (2) long-chain α-neurotoxins (LNTX), which consist of 66–75 residues and five disulfide bridges. In cobras (*Naja* spp.), alpha-neurotoxins are the principal 3FTXs responsible for neurotoxic envenoming, resulting in systemic paralysis and potentially fatal respiratory failure. The abundance of these toxins in venom correlates positively with venom lethality, as measured by intravenous median lethal doses (LD_50_), where higher levels of alpha-neurotoxin protein correspond to greater venom potency [12].

The distribution of alpha-neurotoxins in cobra venom varies across species and geographical regions. Recent findings from cobra venomics and phylogeographical studies reveal a phenotypic dichotomy in venom composition, characterized by a transition in alpha-neurotoxin subtype that accompanies the biogeographical distribution of cobra species, honed by evolutionary pressures to fulfill specific ecological roles. In *Naja naja* (Indian cobra), LNTX predominates, while in Asiatic cobras that have dispersed eastward, a more evolutionarily derived form—without loss of lethality—has largely replaced LNTX. For example, venoms of *N. atra* (Taiwan), *N. kaouthia* (Vietnam), *N. philippinensis*, and *N. samarensis* (Philippines) contain predominantly SNTX, with LNTX virtually absent [12–15]. Despite their structural similarities, SNTX and LNTX exhibit distinct pharmacological profiles at nicotinic acetylcholine receptor (nAChR) subtypes. LNTX is often considered more medically relevant in snakebite envenomation due to its stronger binding affinity toward human nAChRs compared to SNTX [16,17]. This increased affinity is likely due to the additional disulfide bond in loop II of LNTX, which SNTX lacks. Whether this translates to reduced fatality of SNTX in human envenomation is inconclusive, and the toxicity of SNTX should be reckoned with for its high potency and rapid action. Of note, both SNTX and LNTX have comparable lethality in rodent models (LD_50_ values typically range between 0.1 and 0.2 µg/g, intravenously), and the lethality of venom during envenomation depends on the abundance of the dominant alpha-neurotoxin form. For instance, Philippine cobras cause prominent neurotoxicity with rapid onset in humans, despite SNTX being the only alpha-neurotoxin form present in their venom (Watt et al., 1988).

From the treatment perspective, clinically effective antivenoms must be able to immunorecognize and neutralize the dominant neurotoxin in a venom. However, antivenoms often exhibit lower neutralization activity against SNTX than LNTX, possibly due to the limited antigenicity of SNTX proteins [18–20]. SNTX and LNTX share common epitopes within their respective subgroups, enabling cross-reactivity and cross-neutralization by antivenoms raised against different cobra species in Southeast Asia. For instance, *N. kaouthia* Monovalent Antivenom (NkMAV) produced in Thailand can cross-neutralize the venoms of *N. sumatrana* (Equatorial Spitting Cobra, Malaysia) and *N. sputatrix* (Javan Spitting Cobra) [19,21]. However, this is not the case for *N. philippinensis*, as its venom toxicity can only be effectively neutralized by species-specific Philippine Cobra Antivenom (PCAV) [22]. In comparison, the antivenom raised against *N. atra* (Neuro Bivalent Antivenom, Taiwan) shows limited effectiveness against *N. philippinensis* venom. This is unexpected, as proteomic analyses indicate that both species, representing the most eastern-dispersed cobras, predominantly express α-neurotoxins comprising SNTXs, albeit in differing abundances [12,14,23]. These observations raise a broader question of public health importance: Do antigenically distinct “immunoforms” of SNTX exist across Asian cobras, and could this divergence undermine the regional effectiveness of antivenoms used to treat neurotoxic envenoming? Understanding antigenic divergence is directly relevant to snakebite envenoming, a WHO-listed priority neglected tropical disease disproportionately affecting rural communities in Southeast Asia. Regional antivenoms are often relied upon across species and geographical boundaries; however, antigenic incompatibilities may limit cross-neutralization and contribute to treatment failures. While venom composition and sequence variation in cobra toxins have been studied extensively, the degree to which homologous α-neurotoxins differ immunologically remains unclear, particularly for SNTXs, which dominate in Southeast and East Asian cobras.

This study therefore evaluates the immunoreactivity of multiple regional antivenoms toward a panel of elapid venoms and purified α-neurotoxins. We focused on identifying patterns of SNTX antigenicity across species, determining whether distinct immunotypes exist, and characterizing sequence motifs that may underpin divergent antibody recognition. By integrating immunoprofiling with phylogenetic and sequence analyses, we aim to clarify how antigenic divergence among cobra SNTXs shapes antivenom cross-reactivity. These insights may guide the future formulation of antivenoms with broader regional utility and improved therapeutic coverage for neurotoxic snakebite envenomation.

## 2. Materials and methods

### 2.1 Venoms and antivenoms

Venoms of *Naja philippinensis* (Philippines)*, Naja sputatrix* (Indonesia), *Naja naja* (Pakistan), and *Naja kaouthia* (Thailand) were supplied by Latoxan (Valence, France). Venoms of *Naja sumatrana*, *Ophiophagus bungarus* (formerly *Ophiophagus hannah*), *Hydrophis curtus*, and *Hydrophis schistosus* were collected by author (CHT). *Naja atra* venom (Taiwan) was provided by Professor Inn-Ho Tsai from Academia Sinica, Taiwan, while *Laticauda colubrina* venom was from Venom Supplies, Australia. *Calloselasma rhodostoma* venom was provided by the Queen Saovabha Memorial Institute (Bangkok, Thailand). All venom samples were stored in lyophilized form at -20 °C until use.

The alpha-neurotoxins used in this study were previously isolated and characterized in earlier proteomic investigations conducted by the same laboratory, with relevant details summarized in S1 Table. In these studies, toxins were purified by reverse-phase high-performance liquid chromatography (RP-HPLC, Shimadzu LC-20A, Japan) on a C18 column using acetonitrile gradients in 0.1% TFA, and protein purity was verified by analytical re-chromatography and electrospray mass spectrometry, confirming the dominant molecular species corresponding to the annotated database entries. The same well-characterized toxin preparations were utilized in the present immunoreactivity assays. These include SNTXs from *N. philippinensis* [12], *N. sputatrix* [19], *N. kaouthia* [15], *N. atra* [24], *H. curtus* [25], *H. schistosus* [10], and *L. colubrina* [26]. LNTXs were also isolated from venoms of *N. kaouthia* [15], and *O. bungarus* [27], respectively, for immunoreactivity study.

Philippine Cobra Antivenom (PCAV; Lot# 201804) used in the immunoreactivity is a monovalent antivenom raised against *N. philippinensis* and is produced by the Research Institute for Tropical Medicine in the Philippines. *N. kaouthia* Monovalent Antivenom (NkMAV; batch no.: NK00116) is a monospecific antivenom raised against Thai *N. kaouthia* venom, produced by the Queen Saovabha Memorial Institute (QSMI), Bangkok, Thailand. Neuro Bivalent Antivenom (NBAV; batch no.: 61-06-0002) is a bivalent antivenom raised against Taiwanese *N. atra* and *Bungarus multicintus,* manufactured by Taiwan Center for Disease Control, Taipei, Taiwan. Serum Anti Bisa Ular (SABU; batch no.: 4701516) is a polyvalent antivenom raised against *N. sputatrix* (Javan Spitting Cobra), *Bungarus fasciatus* (Banded Krait), and *C. rhodostoma* (Malayan Pit Viper), manufactured by BioFarma Pharmaceuticals, Bandung, Indonesia.

#### Determination of antivenom, venom and isolated toxin concentrations.

Venoms and isolated toxins were quantified at 280 nm using the Nanodrop Spectrophotometer 2000/2000c (ThermoFisher, Rockford, IL, USA), assuming standard protein absorbance behavior. Antivenom concentrations were determined by bicinchoninic acid (BCA) assay according to the manufacturer’s instructions. Venoms and isolated toxins were prepared at a stock concentration of 1 mg/ml for subsequent assays. Protein concentration of Philippine Cobra Antivenom (PCAV), *N. kaouthia* Monovalent Antivenom (NkMAV), Neuro Bivalent Antivenom (NBAV) and Serum Anti Bisa Ular (SABU) were determined using the Thermo Scientific BCA (bicinchoninic acid) Protein Assay Kit (Thermo Scientific Pierce, Waltham, MA, USA). Bovine serum albumin (BSA) was used to obtain a standard curve, according to the manufacturer’s protocol. Antivenom concentrations of samples were determined in three independent experiments, and absorbance values were expressed as means ± S.E.M. The concentrations of PCAV, NkMAV, NBAV and SABU were determined to be 16.2 mg/ml, 57.54 mg/ml, 22.91 mg/ml and 238.76 mg/ml, respectively. Antivenom concentrations were further prepared at a stock concentration of 10 mg/ml and subsequently diluted to 1:450, standardized based on Tan *et al*. [22].

#### Antivenom immunoreactivity study.

The immunoreactivity of Philippine Cobra Antivenom (PCAV), *N. kaouthia* Monovalent Antivenom (NkMAV), Neuro Bivalent Antivenom (NBAV) and Serum Anti Bisa Ular (SABU) were examined with indirect enzyme-linked immunosorbent assay (ELISA) for the above-stated venoms and isolated toxins. For PCAV immunoreactivity, venoms of *N. philippinensis* and *C. rhodostoma* were used as the positive and negative controls, respectively. Positive controls for NkMAV, NBAV and SABU were respective homologous venoms, *N. kaouthia*, *N. atra* and *N. sputatrix*.

In the ELISA experiment, the immunoplate wells were first coated with 10 ng of the respective venoms and isolated toxins (resuspended in the carbonate-bicarbonate coating buffer, pH 9.6) overnight at 4°C. The excess solution was removed by rinsing the wells four times with washing buffer (phosphate-buffered saline with 0.05% Tween-20, pH 7.4). 100 μl of antivenoms (PCAV, NkMAV, NBAV or SABU) at a dilution of 1:450 were added into the venom- or NTX-coated wells, followed by incubation for 1 hr at room temperature. The fixed dilution was selected based on prior optimization experiments to achieve near-saturation binding conditions suitable for comparative antigen-recognition profiling, rather than for quantitative antibody titer determination. The wells were washed four times with washing buffer, flicked dry, followed by addition of horseradish peroxidase (HRP)-tagged secondary antibody (anti-horse HRP-IgG, 1:10000 (Jackson ImmunoResearch Inc. (West Grove, PA, USA) to each well. The mixtures were then incubated at room temperature for another hour and washed four times with wash buffer before adding 50 μl of TMB (3, 3,’ 5, 5’-tetramethylbenzidine) (Merck, Kenilworth, NJ, USA), as the substrate. The enzymatic reaction was allowed to proceed for 25 min at room temperature (25 °C) in the dark, and subsequently terminated with 50 μL of 0.2 M sulfuric acid. The signals developed were read as absorbance at 450 nm using a Tecan M1000Pro Multimode plate reader (TECAN, Switzerland). The immunological binding activities of antivenom to venoms and isolated α-neurotoxins were interpreted by absorbance unit at 450 nm. The assays were performed in three independent experiments, and absorbance values were expressed as means ± S.E.M.

#### Heatmap analysis.

Mean ELISA absorbance values (A₄₅₀) obtained for each antivenom–toxin and antivenom–venom combination were normalized by row z-score transformation and visualized as clustered heatmaps using Perseus (version 2.1.5.0). Clustering of both rows (antivenoms) and columns (venoms or isolated α-neurotoxins) was performed using Euclidean distance and average linkage. The red–white–blue color scale represents relative reactivity, with red indicating stronger and blue weaker binding compared with the mean response of each antivenom.

### Statistical analysis

Values were expressed as means ± SEM of three independent experiments. Differences between absorbance values of isolated alpha-neurotoxins were statistically evaluated using one-way ANOVA with Bonferroni’s *post hoc* test. Results were statistically significant at *p*-value < 0.05.

#### Multiple sequence alignment.

Jalview software (version 2.11.1.3) [28] and the MUSCLE (Multiple Sequence Comparison by Log-Expectation) [29] tool were used to align multiple sequences. Multiple sequence alignment was performed using sequences from related species obtained from the UniProtKB ory (http://www.uniprot.org/) and with reference to transcriptomics reported earlier [30,31]. Other than sequences matched to NTXs experimentally tested in ELISA, homologous SNTX and LNTX sequences were recruited from public databases to provide a broader evolutionary and motif-based context.

#### Phylogeny analysis.

Sequences of short-chain alpha-neurotoxins annotated for Asian elapids (retrieved from Universal Protein Knowledgebase (UniProtKB) (accessed on: January 18, 2025) and transcriptome of *N. sumatrana* [30] were retrieved to construct a phylogenetic tree. The tree was reconstructed using [32] maximum likelihood analysis with bootstrapping (100 replications) in the advanced mode of the phylogeny.fr web server (http://www.phylogene.fr/) (accessed on 20 January 2025), incorporating MUSCLE (v3.7) for multiple alignments, Gblocks (v0.91b) for alignment curation, and PhyML (v3.0) for tree building. Graphical representation and edition of the tree were performed using TreeDyn (v198.3).

### Epitope predictions

Linear B-cell epitopes were predicted using BepiPred-2.0 (threshold = 0.5) and the Emini surface accessibility scale (threshold = 1.0), implemented via the Immune Epitope Database (IEDB) analysis resource. Conformational epitopes were evaluated using DiscoTope 2.0 (threshold = –7.7) based on the corresponding structural models. Four short α-neurotoxins (SNTXs) were selected for epitope analysis, comprising three naturally occurring cobrotoxins from Asiatic cobras: P59276 (*Naja kaouthia*), P80958 and P60770 (*Naja atra*), and one synthetic construct (ScNTX; PDB: 7LUW). These toxins were chosen based on the availability of (i) complete amino acid sequences in the UniProtKB database and (ii) corresponding experimentally determined three-dimensional structures in the Protein Data Bank (PDB), enabling both sequence-based and structure-based epitope prediction.

## Results

### Immunoreactivity of PCAV toward Southeast Asian elapid venoms and isolated alpha-neurotoxins

**Fig 1:**
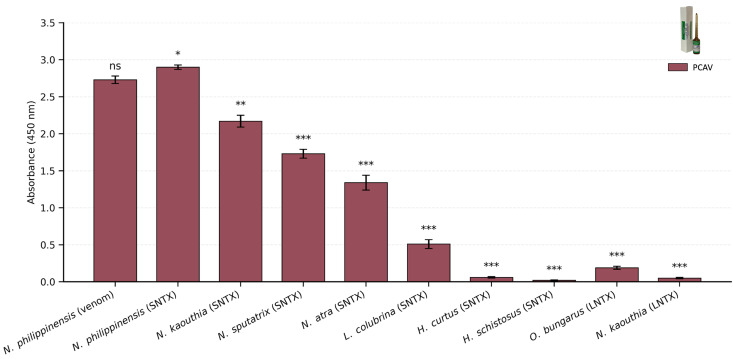
Immunoreactivity of Philippine Cobra Antivenom (PCAV) toward isolated α-neurotoxins (short-chain and long-chain alpha-neurotoxins) of Asian elapids. PCAV binding was measured by indirect ELISA against *N. philippinensis* venom (homologous venom control), short-chain α-neurotoxins (SNTX) from indicated species, and long-chain α-neurotoxins (LNTX). Bars represent mean absorbance at 450 nm ± SEM (n = 3). Statistical significance was assessed by one-way ANOVA with multiple comparisons versus the homologous *N. philippinensis* venom control; ns, not significant; *p* < 0.05, *p < 0.01, **p < 0.001. Abbreviations of genera: *N*: *Naja*, for true cobra; *H*: *Hydrophis*, for sea snake; *L*: *Laticauda*, for sea krait; *O*: *Ophiophagus*, for King Cobra.

PCAV showed varying immunoreactivity toward different short-chain and/or long-chain alpha-neurotoxins (SNTX and LNTX, respectively) isolated from the venoms of four Asian cobras (*N. philippinensis, N. kaouthia, N. sputatrix, and N. atra*), sea snakes (*H. curtus and H. schistocus*), sea krait (*L. colubrina*) and King Cobra (*O. bungarus*) (Fig 1). The highest immunoreactivity was observed in *N. philippinensis* venom (positive control) and its SNTX (abs ≈ 2.7-2.9). The immunoreactivity of PCAV significantly reduced toward the SNTXs of other Asiatic cobras in the following order: *N. kaouthia* (abs ≈ 2.2) > *N. sputatrix* (abs ≈ 1.7) > *N. atra* (abs ≈ 1.4). The immunoreactivity was low toward *L. colubrina* SNTX (abs: ≈ 0.5), and negligible toward the SNTXs of *H. curtus* and *H. schistocus* (abs < 0.1). PCAV also showed minimal immunoreactivity toward the LNTXs of *N. kaouthia* and *O. hannah* (abs ≈ < 0.1). Statistically, the immunoreactivity of PCAV toward most heterologous SNTXs and LNTXs was significantly lower compared to the homologous *N. philippinensis* (one-way ANOVA with multiple comparisons versus control; **p < 0.001)

### Immunoreactivity of regional antivenom products toward Southeast Asian elapid venoms and isolated alpha-neurotoxins

**Fig 2:**
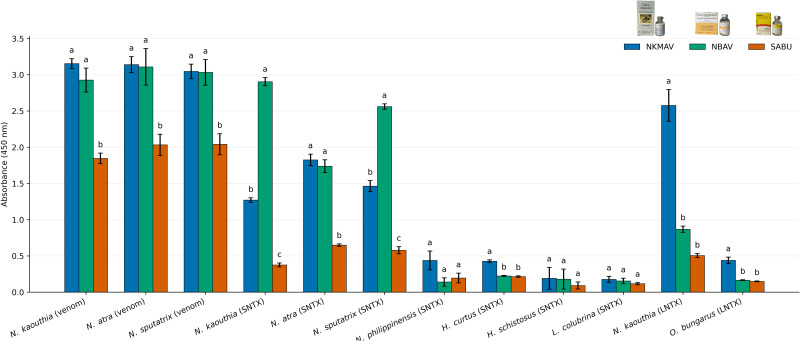
Immunoreactivity of *Naja kaouthia* Monovalent Antivenom (NkMAV), Neuro Bivalent Antivenom (NBAV) and Serum Anti Bisa Ular (SABU) toward venoms and isolated alpha-neurotoxins (SNTX and LNTX) of Asian elapids by indirect ELISA. Values were expressed as means ± SEM from three independent experiments. Abbreviation of genus: *N*: *Naja*, for true cobra; *H*: *Hydrophis*, for sea snake; *L*: *Laticauda*, for sea krait; *O*: *Ophiophagus*, for King Cobra. Values were expressed as means ± SEM from three independent experiments. Different letters indicate significant differences among antivenoms within each venom or toxin antigen group (two-way ANOVA followed by Tukey’s post-hoc test, *p* < 0.05).

Fig 2 shows the comparative immunoreactivity of NkMAV, NBAV and SABU toward the three homologous cobra venoms (*N. kaouthia*, *N. atra*, and *N. sputatrix*, respectively), and alpha-neurotoxins isolated from different elapid species as profiled in Fig 1. NkMAV, raised against the Thai Monocled Cobra, and NBAV, raised against the Taiwan Cobra and Taiwan Krait, showed relatively high immunoreactivity toward the venoms of *N. kaouthia*, *N. atra* and *N. sputatrix* (Abs ≈ 3.0), with no significant difference between the two antivenoms*.* SABU, raised against the Javan Spitting Cobra, Banded Krait and Malayan Pit Viper, had significantly lower immunoreactivity (Abs ≈ 1.8-2.0) toward the three cobra venoms (including its own homologous *N. sputatrix* venom) compared to NkMAV and NBAV (*p* < 0.05).

When tested against isolated SNTX, NkMAV showed significantly lower immunoreactivity (Abs ≈ 1.2-1.5) than NBAV (Abs ≈ 2.5-3.0) toward the SNTXs of *N. kaouthia* and *N. sputatrix*, whereas both antivenoms show comparable immunoreactivity toward the SNTX of *N. atra* (Abs ≈ 1.8). SABU showed significantly lower immunoreactivity than NkMAV and NBAV toward the SNTXs of *N. kaouthia*, *N. atra*, and *N. sputatrix* (Abs ≈ 0.4-0.8). Of note, NkMAV, NBAV and SABU all exhibited low immunoreactivity toward SNTXs of *N. philippinensis* and the marine elapids (*H. curtus*, *H. schistosus*, *L. colubrina*) (Abs < 0.5). Overall, NkMAV was significantly more immunoreactive than NBAV and SABU toward its homologous LNTX (isolated from *N. kaouthia* venom), whereas all three antivenoms showed weak binding to the LNTX of the King Cobra.

### Heatmap analysis and hierarchical clustering

**Fig 3:**
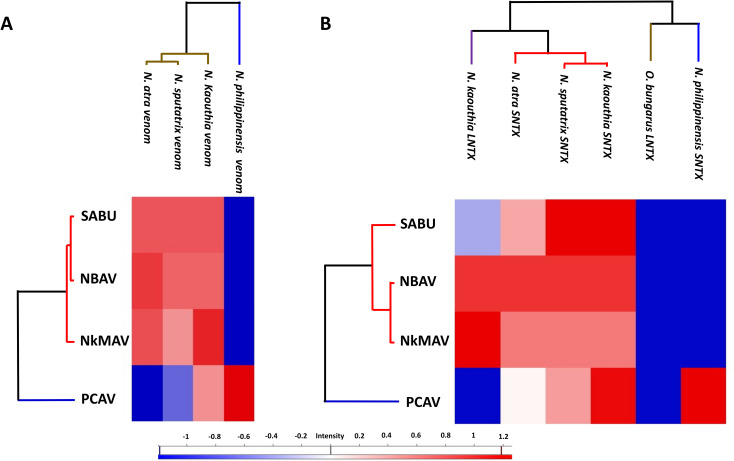
Hierarchical clustered heatmaps showing the immunoreactivity of regional antivenoms toward (A) whole cobra venoms and (B) isolated α-neurotoxins (SNTX and LNTX). Color intensity corresponds to row z-scored ELISA absorbance (A₄₅₀) values, with red indicating higher-than-average and blue lower-than-average binding for each antivenom. Abbreviations: PCAV, Philippine Cobra Antivenom; NKMAV, *Naja kaouthia* Monovalent Antivenom; NBAV, Neuro Bivalent Antivenom; SABU, Serum Anti Bisa Ular; SNTX, short-chain alpha-neurotoxin; LNTX, long-chain alpha-neurotoxin.

Clustered heatmaps (Fig 3) summarized the immunoreactivity profiles of the four regional antivenoms. In the venom panel (Fig 3A), PCAV displayed the strongest homologous binding to *N. philippinensis* venom, whereas NkMAV and NBAV reacted most strongly with *N. kaouthia*, *N. atra*, and *N. sputatrix* venoms. SABU showed generally weaker responses across species. In the toxin panel (Fig 3B), similar clustering patterns were observed: the Philippine cobra SNTX formed a distinct branch separated from the mainland Asian SNTXs, while marine SNTXs and LNTXs from *N. kaouthia* as well as *O. bungarus* grouped independently, reflecting negligible cross-recognition by all antivenoms. Overall, the clustering corroborated the geographical and species specificity of immunoreactivity patterns observed in the ELISA assays.

### Multiple sequence analysis of alpha-neurotoxins

**Fig 4:**
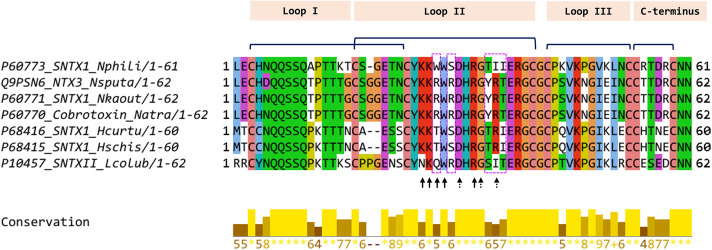
Multiple sequence alignment of short-chain alpha-neurotoxins (SNTXs) from selected elapid species included in the immunoreactivity study. Loops I–III and the C-terminal region are indicated above the alignment. Conserved cysteine residues forming the characteristic disulfide-bonded scaffold of three-finger toxins are shown. Solid arrows indicate primary residues directly implicated in nicotinic acetylcholine receptor (nAChR) binding, whereas dashed arrows denote supporting residues contributing to receptor interaction. The boxed region highlights sequence variability within the receptor-binding motif located in loop **II.** Conservation scores are shown below the alignment.

Amino acid sequences of short alpha-neurotoxins tested for antivenom immunoreactivity were aligned and compared in Fig 4. All SNTX sequences reveal the conserved eight cysteine residues, which result in four disulfide bridges responsible for the folding and stability of a three-finger protein structure. Residues critical for nAChR binding were indicated at the tip of loop II, while residues with high variability and non-consensus within or adjacent to the region were highlighted (in purple boxes). With numbering based on cobrotoxin of *N. atra* (UniProt: P60770, 62 amino acid residues), the 28^th^, 30^th^, 35^th^, 36^th,^ and 37^th^ residues show variability and substitutions. Within the cobra species, two subsets based on the amino acid variation within this region can be recognized, as follows: W28 paired with S30 and ^35^TII^37^, or R28 paired with R30 and ^35^YRT^37^. The corresponding residues in the SNTX of two true sea snakes (*H. schistosus* and *H. curtus*) were conserved (T28, S30, ^35^TRI^37^), whereas in that of the sea krait (*L. laticauda*), the SNTX has Q28, R30, and ^35^SIT^37^ within this peptide region. The K27 residue and residues between the two motifs of the respective subsets, namely ^31^DHRG^34^, remained highly conserved within this critical region of nicotinic receptor binding.

**Fig 5:**
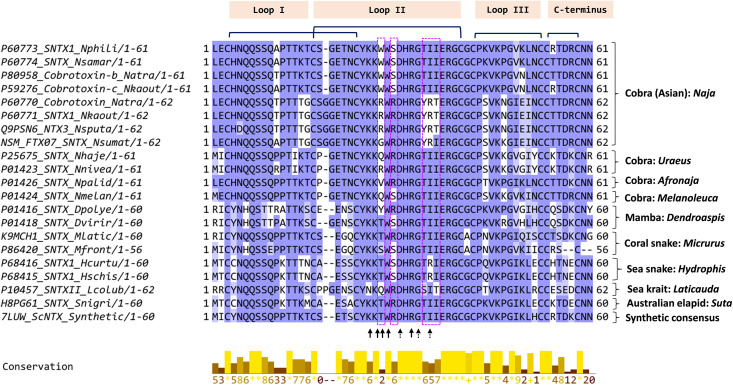
Multiple sequence alignment of representative and database-derived SNTX sequences across elapid lineages including cobras, mamba, coral snake, sea snake, sea krait, Mallee black-back snake (Australian species, *Suta nigriceps*) and a synthetic SNTX (ScNTX) exhibiting the highest similarity to the SNTX of *S. nigriceps*, in addition to experimentally tested SNTXs in immunoreactivity study. Loops I–III and the C-terminal region are indicated above the alignment. Conserved cysteine residues forming the disulfide-bonded scaffold characteristic of three-finger toxins are shown. Residues are shaded according to BLOSUM62 similarity. Solid arrows denote primary residues implicated in nicotinic acetylcholine receptor (nAChR) binding, whereas dashed arrows indicate supporting residues contributing to receptor interaction. The boxed region highlights sequence variability within the receptor-binding motif located in loop **II.** Conservation scores are shown below the alignment.

The amino acid sequences of SNTX from multiple lineages of Elapidae, including cobras (*Naja* spp., along with their subgenera), mambas (*Dendroaspis* spp.), coral snakes (*Micrurus* spp.), sea snakes (*Hydrophis* spp.), sea kraits (*Laticauda* spp.), and the Australian elapid *Suta nigriceps,* as well as the synthetic construct ScNTX, were retrieved from UniProtKB and PDB databases, respectively. The sequences were further aligned for consensus analysis shown in Fig 5. It should be noted that Fig 5 incorporates additional homologous SNTX sequences retrieved from UniProtKB for broader comparative analysis. These additional sequences were not part of the ELISA-based immunoreactivity experiments but were included to illustrate motif conservation and divergence across elapid lineages.

All sequences in Fig 5 retained the characteristic eight-cysteine framework, forming four disulfide bridges that stabilize the three-finger toxin structure. The sequence alignment revealed more variations (specifically, having a lower consensus) in the receptor-binding motif, particularly within loop II, where substitutions were highlighted in purple boxes. Based on numbering relative to *Naja atra* cobrotoxin (P60770, 62 amino acids), key positions of variation were primarily identified at residues 28, 30, 35, 36, and 37, consistent with Fig 4, notwithstanding greater diversity. The two subset motifs shown in Fig 4, i.e., ^28^WWS^30^----35TII^37^, and ^28^RWR^30^----^35^YRT^37^, appear to be consistently present in Asiatic cobras (subgenus: *Naja*) except for the variation noted in *N. sumatrana* (GWR pairing YRI). Here, a schematic overview of the geographic distribution and divergence of representative cobra SNTX motif types is provided in S2 File. On the other hand, the SNTXs of African species exhibit various combination motifs, as in Q/RWR----TII in subgenus *Uraeus*, VWR----TII in subgenus *Afronaja*, and QWS----TII in *Boulengerina*. The African mambas and New World coral snakes too exhibit variation in the two residues (28^th^ and 30^th^ residues based on numbering by P60770); e.g., YWR and TWS in *Dendroaspis* spp., TWR and SWS in *Micrurus* spp. while sharing the conserved TII motif. In the marine elapids, these motifs are more divergent from the terrestrial elapids, where true sea snakes (*Hydrophi*s spp.) show TWS----TRI and sea krait (*Laticauda* sp.) show QWR----SIT. The synthetic ScNTX contains a TWR----TII motif in this corresponding region, and exhibits the highest sequence similarity to the SNTX of *S. nigriceps*, an Australian terrestrial elapid of the Hydrophiinae subfamily in which marine elapids are nested. The overall sequence comparison also reveals conserved N-terminal sequences of SNTX specific to different clades, notable in cobras, where it is typically LECHNQQS in Asiatic cobras and, with minor substitution, MICHNQQS and MECHNQQS in most African cobras. The mambas, New World coral snakes, and Hydrophiinae species show less conserved N-terminus sequence from those of the cobras while retaining the cysteine residue at the 3^rd^ residue position.

**Fig 6:**
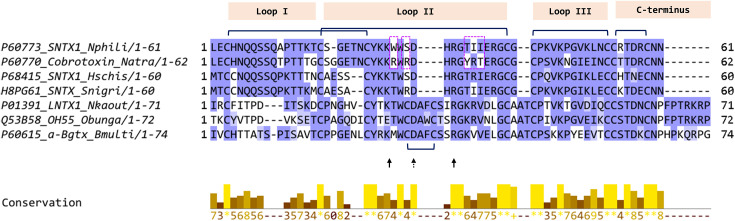
Comparison of representative short-chain neurotoxins (SNTX) and long-chain neurotoxins (LNTX) across elapid lineages, including species whose toxins were experimentally tested in immunoreactivity study. SNTXs from cobras and sea snake are aligned with LNTXs from Monocled Cobra, King Cobra, and Multi-banded Krait. Loops I–III and the C-terminal region are indicated above the alignment. Conserved cysteine residues forming the characteristic disulfide-bonded scaffold of three-finger toxins are shown; note the additional cysteine pair in loop II of long-chain neurotoxins, corresponding to an extra disulfide bond. Residues are shaded according to BLOSUM62 similarity. Solid arrows indicate primary residues implicated in nicotinic acetylcholine receptor (nAChR) binding, whereas dashed arrows denote supporting residues contributing to receptor interaction. Purple boxes highlight sequence variability within the receptor-binding motif of the two cobra SNTXs. Conservation scores are shown below the alignment.

The amino acid sequences of representative short neurotoxins (SNTXs) from representative cobra species and a sea snake were aligned alongside long neurotoxins (LNTXs) from Monocled Cobra (*Naja kaouthia*), King Cobra (*Ophiophagus bungarus*), and Many-banded Krait (*Bungarus multicinctus*) in Fig 6. The sequence alignment highlights conserved features essential for toxin structure and receptor interaction, as well as key regions of divergence that differentiate SNTXs from LNTXs within the three-finger protein family. All sequences maintain the three-finger loop configuration through conserved cysteine framework and disulfide-stabilized three-finger structure (brackets). Of note, the LNTXs contain an additional disulfide bond in loop II, absent in all SNTXs. The loop III and C-terminal regions of LNTXs also show greater divergence, with several charged and hydrophobic substitutions.

A low consensus region marked by differences in the receptor-binding region of loop II is observed, especially within cobra SNTXs, which exhibit two distinct motifs, as described in Figs 3 and 4: ^28^WWS^30^----35TII^37^ (P60773, *N. philippinesis*) and ^28^RWR^30^----^35^YRT^37^ (P60770, cobrotoxin of *N. atra*). In the hydrophiid SNTXs, the motifs are apparently more varied from cobras, e.g., substitutions resulting in TWS----YRI (P68415, *H. schistosus*), and TWR----YII (ACY68680, *S. nigriceps*). LNTXs exhibit greater divergence, including additional hydrophobic and charged residues beyond position 40, as well as an additional disulfide bond, which contribute to a potentially extended receptor interaction site.

### Phylogenetic analysis of short alpha-neurotoxins

**Fig 7:**
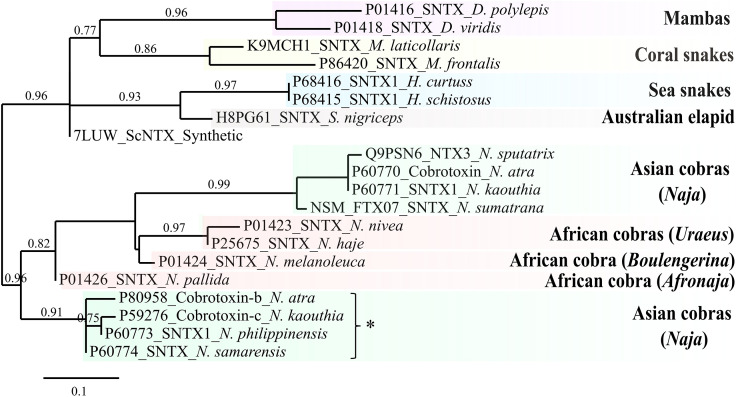
Phylogenetic tree of short neurotoxins (SNTXs) from representative lineages of elapid snakes. The dataset includes mambas (*Dendroaspis polylepsis*, *Dendroaspis viridis*), coral snakes (*Micrurus frontalis*, *Micrurus laticollaris*), sea snakes (*Hydrophis curtus*, *Hydrophis schistosus*), an Australian elapid (*Suta nigriceps*), and true cobras (*Naja* spp.). Cobra sequences comprise Asian cobras of the subgenus *Naja* (*Naja sputatrix*, *Naja kaouthia*, *Naja sumatrana*, *Naja atra*, *Naja philippinensis*, *Naja samarensis*), African cobras of the subgenus *Uraeus* (*Naja nivea*, *Naja haje*), *Boulengerina* (*Naja melanoleuca*), and subgenus *Afronaja* (*Naja pallida*). The sequence 7LUW_ScNTX represents the previously reported synthetic short neurotoxin construct [33]. Sequences marked with an asterisk (*) denote basal position of SNTX variants bearing the distinctive epitope motif “WWS----TII”, in contrast to the derived “RWR----YRT/I” motif found in other Southeast/East Asian cobras. The tree was inferred using the maximum likelihood method implemented in PhyML (v3.1/3.0 aLRT), and visualized using TreeDyn (v198.3).

A maximum likelihood phylogenetic tree was reconstructed to evaluate the evolutionary relationships of short-chain α-neurotoxins (SNTX) across representative elapid taxa, including marine, terrestrial, and arboreal lineages (Fig 7). The analysis reveals well-resolved clades that correspond to extant taxonomic groups, and illustrates the molecular diversification of SNTX sequences within and across the elapid lineages. The SNTXs of sea snakes (*H. curtus* and *H. schistosus*) forms a distinct and well-supported marine clade that is positioned close to the Australian elapid (*S. nigriceps*), whereas the African mambas (*Dendroaspis polylepis* and *Dendroaspis viridis*), and New World coral snakes (*Micrurus* *frontalis* and *Micrurus laticollaris*) form neighboring but separate clusters within the same broader elapid SNTX assemblage. It is worth noting that the hydrophiid (sea snake) lineage (*Hydrophis* spp.) appear divergent from the SNTXs of sampled elapids, indicating a distinct evolutionary trajectory for their SNTX. This, however, does not imply that sea snakes are more “primitive”; rather, their SNTX sequence placements reflect divergence from that of a common elapid ancestor before the diversification of the terrestrial cobra-mamba-coral snake lineage. Their evolution as marine specialists suggests subsequent adaptation rather than ancestral retention of features.

In the phylogenetic analysis, the synthetic short neurotoxin construct (7LUW_ScNTX) was introduced into the tree for comparison and was found positioned outside the main *Naja* radiations, resolving closer to the node associated with the assemblage comprising sea snakes (*Hydrophis*), the Australian elapid (*Suta*), mambas (*Dendroaspis*), and coral snakes (*Micrurus*). The true cobras (*Naja*) formed a distinct clade and were further partitioned into major lineages, broadly consistent with their geographic and subgeneric divergence. Within this cobra clade, the Asian cobras of the subgenus *Naja* showed further differentiation, including a small basal assemblage (marked with an asterisk) which comprises sequences from *N. philippinensis* and *N. samarensis*, together with selected SNTX variants from other Asian cobras.

### Epitope prediction of short alpha-neurotoxins

**Fig 8:**
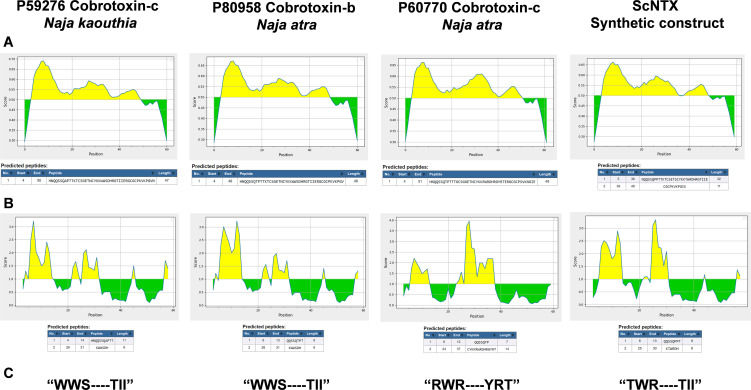
Linear epitope prediction of SNTXs. Linear B-cell epitope propensity and surface accessibility of four short α-neurotoxins (SNTXs): P59276 (*Naja kaouthia*), P80958 and P60770 (*Naja atra*), and ScNTX (synthetic construct), were predicted using the IEDB analysis resource. **(A)** BepiPred-2.0 (threshold = 0.5). **(B)** Emini surface accessibility scale (threshold = 1.0). Predicted epitope regions are highlighted in yellow. **(C)** Loop II motif variants (WWS----TII, RWR----YRT, TWR----TII) associated with antigenic divergence.

Fig 8 shows broadly similar linear epitope profiles among the four SNTXs, with all toxins displaying a dominant antigenic region spanning the N-terminal half to central portion of the sequence in the BepiPred-2.0 analysis (Fig 8A), together with additional surface-accessible regions in the Emini plot (Fig 8B), and motif variants in loop II (Fig 8C). Notably, the two SNTXs bearing “WWS----TII” motif in loop II, i.e., P59276 from *N. kaouthia* and P80958 from *N. atra*, show highly similar profiles in both the extent and continuity of their predicted linear epitopes, supporting relative conservation of antigenic features despite originating from different species. In contrast, the SNTX with a “RWR----YRT” motif (P60770) from *N. atra* exhibits a shifted and somewhat more segmented surface accessibility pattern, with stronger central reactivity and a distinct reduction in the N-terminal accessible region, suggesting altered local antigenic presentation associated with the “RWR----YRT” motif class. The synthetic construct ScNTX, which contains the intermediate TWR----TII motif, shows a mixed profile: its BepiPred pattern remains broadly similar to the native toxins, whereas its Emini plot more closely resembles the extended and discontinuous accessibility pattern seen in P60770. Together, these results suggest that SNTXs bearing “WWS----TII” motif retain a more conserved linear antigenic architecture, whereas substitution toward RWR----YTR is associated with measurable changes in predicted epitope continuity and surface exposure, consistent with emerging antigenic divergence between the two motif classes.

**Fig 9:**
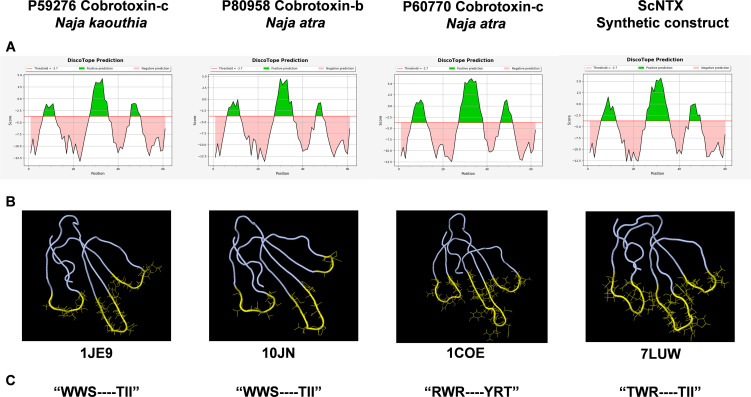
Structure-based epitope prediction of SNTXs. Structure-based epitope prediction of the same four SNTXs using DiscoTope 2.0 and corresponding PDB structures (1JE9, 1ONJ, 1COE, and 7LUW). **(A)** DiscoTope scores (threshold = –7.7; red line), with positive residues shown in green. **(B)** Structural mapping of predicted epitope residues (yellow) on toxin models in Jmol mode. **(C)** Loop II motif variants (WWS----TII, RWR----YRT, TWR----TII) associated with antigenic divergence.

Fig 9 A-B shows structure-based epitope predictions that further distinguish antigenic features between “WWS----TII” and “RWR----YRT” motif classes (Fig 9C). In Fig 9A, DiscoTope analysis indicates that all SNTXs possess dominant conformational epitopes in loop II; however, the “WWS----TII” toxins (P59276 and P80958) exhibit relatively confined high-scoring regions centered at the loop II apex, whereas the “RWR----YRT” toxin (P60770) displays a broader and more continuous distribution of positive scores extending along loop II and in loop **III.** The synthetic construct (ScNTX; TWR----TII) shows an intermediate profile, retaining a prominent loop II epitope while exhibiting extended continuity similar to the “RWR----YRT” toxin. These differences are further reflected structurally in Fig 9B, where epitope mapping reveals localized exposure at the loop II tip in “WWS----TII” toxins, in contrast to the more extensive surface distribution across loop II and adjacent regions, including loop III, in P60770 and ScNTX. Together, these findings indicate that substitution from WWS----TII to RWR----YRT motifs is associated with expansion and redistribution of conformational epitopes, supporting a structural basis for antigenic divergence among SNTX variants.

## Discussion

Alpha-neurotoxins (α-NTXs), comprising long-chain (LNTX) and short-chain (SNTX) neurotoxins, are functionally conserved snake venom proteins that show potent antagonistic activity toward muscle-type nicotinic acetylcholine receptors (nAChRs) [34]. While this function appears to have evolved convergently across multiple elapid lineages as an adaptation for predation, α-NTXs are evolutionarily labile. Positive selection, driven primarily by past and perhaps ongoing evolutionary arms race, subjects these proteins to accelerated evolution [8]. This evolutionary dynamic has led to substantial sequence diversification, particularly at surface-exposed residues across elapid species, while preserving the structural framework essential for receptor binding. In comparison to SNTXs, LNTXs have traditionally received more attention owing to their higher binding affinity toward human muscle nicotinic receptor (nAChR) and thus, a “perceived” higher risk of envenomation in human [17]. Nonetheless, SNTXs are increasingly recognized for their limited immunogenic capacity (in comparison to LNTXs) [3,19,33], and the high abundance in the venoms of some Asian *Naja* species, particularly those from Southeast and East Asia, simply cannot be overlooked [12–14,19]. Previous studies attributed the poor immunogenicity of SNTX to its small size (~60–62 amino acids), an inherent factor limiting epitope display for effective immune response [20,35]. Beyond the molecular size of the SNTX, in this study, we showed that SNTXs display subtle sequence-level variations that may critically influence their antigenicity and thus, impacting the effectiveness of antivenom therapy. As revealed in a series of Asiatic cobra venomics, there is a transition from LNTX- to SNTX-dominant phenotype as cobras diversified from the west to the east, with the easternmost dispersal lineage of *Naja* spp., represented by *N. philippinensis* and *N. samarensis* of the Philippines, characterized by extremely lethal venoms containing exceptionally high abundance of SNTXs (44–66%) with no LNTXs [12,13]. The same is true for *N. atra*, albeit with a lower abundance of SNTX (~10%) and lower lethality [14,23]. Intriguingly, the Philippine spitting cobra venom could not be effectively cross-neutralized by antivenom raised against *N. atra*, suggesting antigenic variation between the two. On the other hand, the venoms of other Asian *Naja* spp. had lower abundances of SNTXs, typically ranging from 1-10% of total venom protein (Table 1). The remarkable variation in both abundance and epitope composition of SNTXs between the Philippine species and other Asian cobras may explain the reduced immunoreactivity of PCAV toward venoms and isolated SNTX of these Southeast Asian cobras.

**Table 1:** Composition of short- and long-chain alpha-neurotoxins from representative Afro-Asian cobras (*Naja* spp.), and marine elapid species. Values represent the percentage abundance of short-chain and long-chain α-neurotoxins relative to total venom protein, as reported in the cited quantitative proteomic studies. Ranges indicate values reported from different geographical populations or independent studies. ND, not detected.

Species	Alpha-neurotoxins (% of total venom protein)		Reference
	Short-chain	Long-chain	
**Asian cobras**			
**Subgenera *Naja***			
*Naja samarensis*	65.87	ND	[36]
*Naja philippinensis*	44.55	ND	[37]
*Naja siamensis*	5.78	2.83	[24]
*Naja sputatrix*	7.89	0.48	[19]
*Naja sumatrana*	2.79-6.41	2.62-10.30	[21,38]
*Naja naja*	0.86	9.61	[39]
*Naja atra*	10.30	1.34	[14]
*Naja* (Thailand)	7.70	33.30	[40]
*Naja karachiensis*	8.00	24.00	[41]
**African cobras**			
**Subgenera *Boulengerina***			
*Naja melanoleuca*	9.30	13.50	[42]
**Subgenera *Afronaja***			
*Naja nubiae*	12.60	ND	[43]
*Naja pallida*	2.80	ND	
*Naja nigricollis*	0.40	ND	
*Naja mossambica*	1.60	ND	
*Naja katiensis*	4.40	ND	
**Subgenera *Uraeus***			
*Naja haje*	34.45	34.61	[24]
*Naja senegalensis*	6.47	16.26	[44]
*Naja nivea*	3.61	3.78	[45]
*Naja annulifera*	3.56	0.16	[46]
**True sea snakes**			
*Hydrophis schistosus*	55.80	14.70	[10]
*Hydrophis curtus*	22.89	3.44	[31]
**Sea krait**			
*Laticauda colubrina*	16.94	48.87	[26]

Based on the previous study evaluating the neutralizing activity of Philippine Cobra Antivenom (PCAV) against the venoms of *Naja samarensis* and *Naja philippinensis* [22], the present immunoreactivity findings confirm that these far-eastern, insular cobras endemic to the Philippines possess SNTXs with distinct antigenic profiles, differentiating them from other Southeast Asian cobras. Notably, PCAV showed markedly reduced cross-reactivity toward SNTXs from non-Philippine cobra species, following the order: *N. kaouthia* > *N. sputatrix* > *N. atra*. This pattern may reflect historical biogeographic dispersal, in which the Philippine cobra species could have descended from an ancestral lineage in continental Asia that radiated southward and eastward, with *N. kaouthia* and *N. sputatrix* representing intermediate lineages, while *N. atra* diverged along an eastward trajectory into mainland China approximately 1–2 Mya, and subsequently reached Taiwan during the Late Pleistocene glacial periods (~20,000–70,000 years ago) before the submersion of the land bridge. In Taiwan’s insular and montane environments, further ecological adaptation in *N. atra* might have resulted in a venom phenotype that includes SNTXs with antigenic features that are much more divergent from their Philippine counterparts. In fact, even within the mountainous island of Taiwan, the Central Mountain Range was speculated to be a biogeographic barrier that resulted in two variable phenotypes between the eastern and western *N. atra* populations, as reported in both their morphology and venom compositions [14,47]. The present study also confirmed the very limited cross-reactivity of PCAV toward SNTXs of marine elapids, including sea kraits and sea snakes, as well as LNTXs from both *N. kaouthia* (a true cobra) and *O. bungarus* (King Cobra) in Southeast Asia due to greater variation in their sequences and epitopes.

We also further evaluated the immunorecognition of αNTXs by other cobra antivenoms available in the region: *N. kaouthia* Monovalent Antivenom (NkMAV, Thailand), Neuro Bivalent Antivenom (NBAV, Taiwan), and Serum Anti Bisa Ular (SABU, Indonesia). The present ELISA design employed a fixed, near-saturating antivenom dilution to compare relative antigen recognition patterns rather than quantitative antibody titers or binding affinities. NkMAV showed significantly higher immunological binding activity among the antivenoms toward LNTXs of *N. kaouthia*, which is intuitively appropriate as the Thai *N. kaouthia* venom is used as its immunizing venom. Furthermore, in comparison to various other southeast Asian cobras including its own species from other geographical locales, the Thai *N. kaouthia* venom contains the highest abundance of LNTXs (35% of total venom protein) [15]. Hypothetically, NkMAV is an LNTX-targeting antivenom for cobra species within the region, e.g., *N. sumatrana,* which also shares a similar LNTX-dominant venom phenotype. Indeed, NkMAV is used clinically as the antivenom treatment in Malaysia where *N. kaouthia* and *N. sumatrana* are both found [48], with success to some extent. However, NkMAV is not not indicated for envenoming by King Cobra (*O. hannah* and *O. bungarus*) in spite of its LNTX-dominant venom phenotype, since King Cobra’s and *Naja* cobra’s LNTXs are evolutionarily and antigenically diverged [49]. While exhibiting high immunoreactivity toward the *N. kaouthia* LNTX, NkMAV consistently showed a lower immunoreactivity by at least 30% toward the SNTXs of its own and heterologous *N. sputatrix* as well as *N. atra*, implying that an “LNTX-targeting” antivenom, as proposed above, has weak binding activity for SNTX of these cobras. On the other hand, NBAV, raised against Taiwan Cobra venom, which contains SNTX as its sole alpha-neurotoxin [14,23], exhibited higher binding activities toward these SNTXs. Meanwhile, NBAV’s binding activity toward the LNTX is, unsurprisingly, significantly lower than NkMAV. The finding further supports that LNTX and SNTX are indeed antigenically variable, with the latter (SNTX) having a lower immunogenicity than LNTX [20]. The Indonesian SABU antivenom, however, had the overall lowest immunoreactivity toward all alpha-neurotoxins, a finding consistent with its polyvalent (trivalent) nature (having the smallest portion of SNTX from *N. sputatrix*), and its lower content of immunoglobulins, immunological binding activity as well as neutralization efficacy [50]. Finally, a finding that prompted the question of antigenic divergence in the SNTXs of southeast Asian cobras: all three antivenoms (NkMAV, NBAV, SABU) lacked immunoreactivity toward the SNTXs of *N. philippinensis* and marine elapids, highlighting species-specific and biogeographically influenced epitope variation in cobra SNTXs. The lack of immunoreactivity suggests limited cross-neutralization potential; however, establishing direct *in vivo* correlations would require systematic cross-neutralization studies in animal models. Given the low cross-reactivity observed among several heterologous antivenoms, these experiments were not pursued, and the present work instead focuses on mechanistic insights into antigenic divergence at the molecular level.

The heatmap visualization highlights the antigenic divergence between the Philippine and other Asian cobra neurotoxins. The strong homologous binding of PCAV to *N. philippinensis* SNTX contrasts with the limited cross-recognition by other regional antivenoms, supporting the notion that short-chain α-neurotoxins have evolved with geographically distinct antigenic epitopes. NkMAV and NBAV clustered closely, indicating shared recognition of major SNTXs from *N. kaouthia*, *N. atra*, and *N. sputatrix*, whereas all antivenoms showed weak recognition of long-chain α-neurotoxins, particularly *O. bungarus* LNTX. The finding implies that current immunization mixtures primarily reflect the SNTX antigenic profiles of regional cobras, leading to restricted cross-neutralization, and reinforces the need to incorporate representative antigens from both SNTX motif lineages to broaden antivenom coverage.

Although SNTXs constitute the dominant lethal component in the venoms examined, whole-venom neutralization in clinical settings is inherently multifactorial. Minor venom constituents may synergize to enhance toxicity and influence the overall immunogenic profile of the venom used for antivenom production. The present study focuses on isolated SNTX fractions to dissect subtype-specific antigenic variation, while acknowledging that this reductionist approach may not fully recapitulate the complexity of whole-venom interactions during envenoming or immunotherapy. Here, given that the observed antigenic divergence most plausibly arises from epitope variability among SNTXs, we further aligned and analyzed the SNTX sequences to determine whether specific motif-level differences might serve as drivers of antigenic subclasses. Rather than modeling every experimentally tested toxin individually, structurally characterized SNTXs carrying either the “WWS–TII” or “RWR–YRT” motif were chosen as representatives to evaluate whether these motif-level differences may translate into altered antigenic surface exposure. The synthetic scNTX sequence, previously designed to represent a consensus short-chain α-neurotoxin construct, was included as a comparative reference in sequence alignment and *in silico* epitope analyses. The sequence analysis reveals two dominant loop II motifs in Asian cobras: “^28^WWS----TII^37^” and “^28^RWR----YRT^37^”, between which the sequence “^31^DHRD^34^” remained highly conserved across various lineages. *N. philippinensis* and *N. samarensis* expressed SNTXs with only the “^28^WWS----TII^37^” motif, whereas *N. atra* (Taiwan) and *N. kaouthia* (Thailand) express two forms of SNTXs, each contains “^28^WWS----TII^37^” and “^28^RWR----YRT^37^”, respectively, with the latter form “^28^RWR----YRT^37^” being the majority. The *N. sputatrix* (Indonesia) SNTX contains only the “^28^RWR----YRT^37^” form of SNTX, while that of *N. sumatrana* (Malaysia) in this study revealed a variation in this motif as “^28^GWR----YRI^37^”. The 28^th^ glycine (^28^G) or arginine (^28^R) residue has a different property from the tryptophan residue (^28^W) which contains a large hydrophobic, double-ring aromatic side chain. As glycine is small, the GWR motif may simply unmask or provide flexibility to the bulky WR pair next to it, maintaining a similar shape to the “^28^RWR----YRT^37^” motif of *N. sputatrix*, which is also shared between *N. kaouthia* (Thailand) and *N. atra* (Taiwan) as their major form of SNTX. These dichotomous motifs agree with the variable immunoreactivity of PCAV that binds effectively to SNTX containing the motif “^28^WWS----TII^37^” but not “^28^RWR----YRT^37^”, while the reverse is true for NBAV, NKMAV, and SABU (i.e., binding effectively to SNTX having the motif ^28^RWR----YRT^37^” but not “^28^WWS----TII^37^”. The finding suggests an epitope-level divergence between the two motifs in these cobra SNTXs despite sharing relatively close phylogenetic relatedness and biogeographic proximity in Southeast Asia and East Asia. On the other hand, the SNTXs of African cobras vary from those of the Asiatic species to a much greater extent, whereby some of the 28^th^ amino acid residues are substituted by glutamine (Q) or valine (V) while retaining the conserved region of ^29^W and ^30^R/^30^S residues within the motif. The African mambas, New World coral snakes, and hydrophiid species have an even more variable combinations of the motif (^28^XWX^30^) within this critical region of loop II, further indicating that although this functional region is broadly conserved for nAChR binding [16], surface-exposed residues in and around the loop can mutate and diverge antigenically. Ostensibly, the mutation begat variable epitopes which brought advantage in a predator-prey arms-race, as these cobras of different species adapt to distinct ecological niches throughout Asia and Africa. Interestingly, the synthetic construct (ScNTX) harbors the motifs of “^28^TWR^30^” and “^35^TII^37^” (along with the all-conserved “^31^DHRG^34^” motif) in its loop II, which are, by sequence comparison, highly similar (nearing 100%) to the SNTX of an exotic and endemic terrestrial elapid, *S. nigriceps* from Australia. Its “^28^TWR^30^” motif, in comparison to the abovementioned “^28^RWR^30^” and “^28^WWS^30^” motifs of Asiatic cobras’ SNTXs, deserves a note of distinction in terms of their structure and antigenicity. Both the constructed “^28^TWR^30^” and the native “^28^RWR^30^” contain tryptophan (W) and arginine (R) at positions 29 and 30, with only one residue difference, *i.e.*, threonine (T) *vs.* arginine (R) at position 28. Threonine is smaller and polar, while arginine is larger and basic. Still, the difference is not as drastic as introducing a tryptophan (W) as in the case of the “^28^WWS^30^” motif, which contains two bulky tryptophan residues—this could alter the local conformation significantly. Also, its 30^th^ residue is substituted by serine (S), an uncharged polar residue, unlike the basic (typically also more antigenic) arginine (R) at position 30. Hence, both “^28^TWR^30^” and “^28^RWR^30^” should have a higher potential to form a similar antigenic surface. At the same time, the “^28^WWS^30^” motif contributes to a surface with higher hydrophobicity and lower charge, resulting in lower antigenic similarity to the other two and ultimately facilitating antigenic escape.

On the other hand, the secondary variable motif “^35^TII^37^” is conserved with that of the SNTXs carrying “^28^WWS^30^” motif (notably in *N. philippinensis* and *N. samarensis*, and in African cobra species). Comparing between “^35^TII^37^” and “^35^YRT^37^” motifs (the latter is paired with the “^28^RWR^30^” motif, predominantly found in *N. kaouthia*, *N. sputatrix,* and *N. atra*), the ^35^TII^37^ motif likely contributes to protein core structure or membrane interactions rather than immunorecognition, as this relatively compact region is mostly hydrophobic with low surface exposure for antibody accessibility. Further sequence comparison between representative short neurotoxins (SNTXs) from *N. philippinensis*, *N. atra*, *H. curtus*, and *S. nigriceps*, and long neurotoxins (LNTXs) from *N. kaouthia*, *O. bungarus* (*O. hannah*), and *B. multicinctus* revealed greater divergence in residues flanking the critical sites. Notably, in the LNTXs analyzed, the 30^th^ residue is replaced by a cysteine, creating motifs such as ^28^TWC^30^ or ^28^MWC^30^. This substitution enables the formation of an additional loop, stabilized by a disulfide bond between the cysteine residues at positions 30 and 35. This innovative structural role has a functional implication: a conformational constraint is introduced to stabilize the local structure of loop II, modulating the surface topology and possibly enhancing binding affinity to muscle-type nicotinic acetylcholine receptors (nAChRs) through greater hydrophobic and electrostatic contacts [51].

To place the observed loop II motif divergence into an evolutionary context, we reconstructed the phylogenetic relationships of the analyzed SNTXs. The sequences cluster within the classical type I (short-chain) α-neurotoxin lineage of the three-finger toxin superfamily, consistent with established evolutionary classifications of elapid 3FTxs [52]. Within this lineage, the two major loop II motif classes identified in this study form distinct sub-clusters, suggesting that antigenic divergence corresponds with evolutionary branching rather than random residue variation. The integration of the synthetic sequence provides a comparative anchor to assess the evolutionary polarity of natural sequence changes, especially in structurally and immunologically relevant regions with epitope recognition and receptor binding. The tree supports lineage-specific diversification of SNTXs within the genus *Naja*, with antigenic divergence accompanying geographic and subgeneric separation. Within the Asian cobra clade (subgenus *Naja*), the basal SNTX variants are characterized by the distinctive, unique epitope motif “WWS----TII”, whereas the more derived SNTXs predominantly carry the divergent motif “R/GWR----YRT/I”. This topology supports the view that the “WWS----TII” motif represents an earlier SNTX form within the Asian cobra lineage, from which the more antigenically divergent “R/GWR----YRT/I” variants subsequently emerged. The exclusive presence of the basal motif in the Philippine cobras, *N. philippinensis* and *N. samarensis*, further suggests retention of an ancestral-like epitope configuration in these endemic, insular species, whereas *N. atra*, *N. kaouthia*, and other Asiatic cobras appear to have shifted toward and prioritized the derived form.

Building on this evolutionary framework, the epitope prediction analyses provide structural and functional context for the observed loop II motif divergence. Across both linear and structure-based predictions, loop II consistently emerges as the dominant antigenic region, in line with its established role in receptor binding. However, clear differences are evident between motif classes. The “WWS----TII” toxins exhibit a more confined and localized epitope profile, largely centered at the apex of loop II, whereas the “RWR----YRT” toxin shows a broader and more continuous antigenic surface extending along loop II and in loop III, accompanied by increased surface accessibility. This expansion is recapitulated by the synthetic construct (TWR----TII), which displays an intermediate but more extended epitope architecture. Together, these findings suggest that the evolutionary transition from the ancestral WWS----TII motif to the derived RWR----YRT motif is associated with a shift from localized to more distributed antigenic surfaces, potentially enhancing epitope exposure and variability. Such structural redistribution of antigenic determinants provides a plausible mechanistic basis for the antigenic divergence observed among SNTXs, and may contribute to differences in antibody recognition and cross-reactivity across cobra lineages.

The findings indicate that motif-level differences represent fine-scale diversification within a conserved structural scaffold and parallel the differential immunoreactivity patterns observed experimentally. Overall, the notable variation at positions 28, 30, and 35 occurring within structurally tolerant regions of the cobra alpha-neurotoxins is in line with the “Three-Fingered RAVER” theory (rapid antigenic variation in exposed residues) in the three-finger toxin superfamily [8]. The variation reflects accelerated evolution as an adaptive strategy to diversify antigenicity without compromising receptor binding or venom potency. The identification of divergent loop II antigenic motifs through sequence alignment and in silico epitope prediction tools provides a coherent, robust theoretical framework that aligns with the consistent immunoreactivity patterns observed in this study. While high-resolution structural characterization of antibody-toxin interfaces or targeted mutational analyze would be required to establish a definitive causal relationship between specific residues and antibody escape, the proposed epitope-driven mechanism should be interpreted as a mechanistically supported model rather than conclusive molecular proof.

Considering the high degree of evolvability in this class of toxins, the pursuit of a universal anti-SNTX antivenom that is ubiquitously effective against all SNTX types deserves further and thorough investigation, including the search for non-antibody-based alternatives such as small-molecule inhibitors and *de novo* designed mini-binders of venom proteins [53,54]. Nonetheless, antivenoms will likely remain the cornerstone of therapy for the foreseeable future. Therefore, optimizing antivenom formulations should involve incorporating a balanced repertoire of anti-LNTX and anti-SNTX antibodies, taking into account not only the structural subtypes of 3FTX toxins but also their relative abundance within the venom proteome, as well as potential epitope variations that may occur even among structurally conserved toxins across geographically distinct species.

### Conclusion

This study offers a new perspective on the divergence of cobra venom short-chain alpha-neurotoxins (SNTXs), which exhibit significant antigenic variability despite structural conservation. The Philippine spitting cobras (*N. philippinensis* and *N. samarensis*) express SNTXs with an ancestral-like loop II motif (^28^WWS–TII^37^), which are strongly recognized by PCAV but poorly by other regional antivenoms. In contrast, Asiatic cobras such as *N. kaouthia*, *N. atra*, and *N. sputatrix* predominantly express a divergent and more derived form of the corresponding motif (^28^RWR–YRT^37^), which may contribute to the limited cross-recognition observed for PCAV. Conversely, antivenoms raised against the ^28^RWR–YRT^37^ type of SNTX (predominant forms in *N. kaouthia*, *N. atra*, *N. sputatrix*) showed minimal binding toward the ^28^WWS–TII^37^ type of SNTX from the Philippine cobras, suggesting limited cross-neutralization potential. These findings underscore that antigenicity differences at key epitope residues, rather than size constraints alone, contribute to the poor recognition of SNTXs. From a clinical perspective, this divergence highlights the challenges of developing a universal anti-cobra antivenom. Together, our findings highlight that SNTX antigenicity is shaped by discrete sequence motifs, which define regional immunoreactivity barriers. Rational antivenom development in the future should integrate these antigenic subtypes alongside LNTXs to broaden antivenom cross-species efficacy.

## Supporting information

S1 Table Short and long alpha-neurotoxins and respective species used in the immunoreactivity study. (PDF)

S2 File Geographic distribution and antigenic divergence of cobra short-chain α-neurotoxins (SNTXs) associated with species-specific antivenom recognition in Southeast and East Asia. (PNG)
