# Supplementary material for: Antigenic divergence of cobra short-chain α-neurotoxins: Implications for regional antivenom effectiveness in Southeast Asia
Source: PLoS Negl Trop Dis. 2026 May 14;20(5):e0013859. doi: 10.1371/journal.pntd.0013859 (PMC13268184; doi:10.1371/journal.pntd.0013859)
Supplement: S1 Table — (PDF) [file pntd.0013859.s001.pdf]

Supporting information:

S1 Table. Short and long alpha-neurotoxins and respective species used in the immunoreactivity study. The proteins were previously isolated through C18 reverse-phase high-performance liquid chromatography (Shimadzu LC-20AD, Shimadzu, Tokyo, Japan) from the same laboratory [1-8].

**Species**	**Protein subtype**	**Accession number**	**Protein annotation**	**References**
Naja philippinensis	SNTX	P60773	Short neurotoxin 1	Tan et al. [1]
Naja sputatrix	SNTX	Q9PSN6	Neurotoxin 3	Tan et al. [2]
Naja kaouthia	SNTX	P60771	Short neurotoxin I	Tan et al. [3]
Naja atra	SNTX	P60770	Cobrotoxin	Wong [4]
Hydrophis curtus	SNTX	P68416	Short neurotoxin 1	Tan et al. [5]
Hydrophis schistosus	SNTX	P68415*	Short neurotoxin 1	Tan et al. [6]
Laticauda colubrina	SNTX	P10457	Short neurotoxin II	Tan et al. [7]
Naja kaouthia	LNTX	P01391	Alpha-elapitoxin-Nk2a	Tan et al. [3]
Ophiophagus bungarus	LNTX	Q53B58	Long neurotoxin OH	Diew [8]

Note: Toxin peaks were purified by RP-HPLC and identified by mass spectrometry. These are alpha-neurotoxins including homologous SNTXs from *N. philippinensis* [1], *N. sputatrix* [2], *N. kaouthia* [3], *N. atra* [4], *Hydrophis curtus* [5], *Hydrophis schistosus* [6], and *Laticauda colubrina* [7]. Short neurotoxin of *H. schistosus* was taken as P68415 in the current study based on its homology to neurotoxin A (P01437) of *Hydrophis lapemoides*, which was annotated in [6]. LNTXs were also isolated from the venoms of *N. kaouthia* [3], and *Ophiophagus bungarus* [8], respectively, for the immunoreactivity study. Abbreviation: SNTX: Short-neurotoxin; LNTX: Long-neurotoxin.

References

1. Tan CH, Wong KY, Chong HP, Tan NH, Tan KY. Proteomic insights into short neurotoxin-driven, highly neurotoxic venom of Philippine cobra (*Naja philippinensis*) and toxicity correlation of cobra envenomation in Asia. J Proteomics. 2019;206:103418.
2. Tan NH, Wong KY, Tan CH. Venomics of *Naja sputatrix*, the Javan spitting cobra: A short neurotoxin-driven venom needing improved antivenom neutralization. Journal of Proteomics. 2017;157:18-32.
3. Tan KY, Tan CH, Fung SY, Tan NH. Venomics, lethality and neutralization of *Naja kaouthia* (monocled cobra) venoms from three different geographical regions of Southeast Asia. J Proteomics. 2015;120:105-25.
4. Wong KY. Proteomics and Toxinological Study of the Venoms from Afro-Asian Cobras (*Naja* spp.). Kuala Lumpur, Malaysia: University Malaya; 2019.
5. Tan CH, Tan KY, Ng TS, Sim SM, Tan NH. Venom proteome of spine-bellied sea snake (*Hydrophis curtus*) from Penang, Malaysia: Toxicity correlation, immunoprofiling and cross-neutralization by sea snake antivenom. Toxins. 2019;11:3.

6. Tan CH, Tan KY, Lim SE, Tan NH. Venomics of the beaked sea snake, *Hydrophis schistosus*: A minimalist toxin arsenal and its cross-neutralization by heterologous antivenoms. J Proteomics. 2015;126:121-30.
7. Tan CH, Wong KY, Tan KY, Tan NH. Venom proteome of the yellow-lipped sea krait, *Laticauda colubrina* from Bali: Insights into subvenomic diversity, venom antigenicity and cross-neutralization by antivenom. J Proteomics. 2017;166:48-58.
8. Lau DF. Toxinological Characterization of Long Neurotoxin from Malaysian King Cobra (*Ophiophagus hannah*) Venom. Kuala Lumpur, Malaysia: University Malaya; 2019.

Legend

S1 Table. Short and long alpha-neurotoxins and respective species used in the immunoreactivity study.
