# Supplementary figures and images for: Antigenic divergence of cobra short-chain α-neurotoxins: Implications for regional antivenom effectiveness in Southeast Asia
Source: PLoS Negl Trop Dis. 2026 May 14;20(5):e0013859. doi: 10.1371/journal.pntd.0013859 (PMC13268184; doi:10.1371/journal.pntd.0013859)

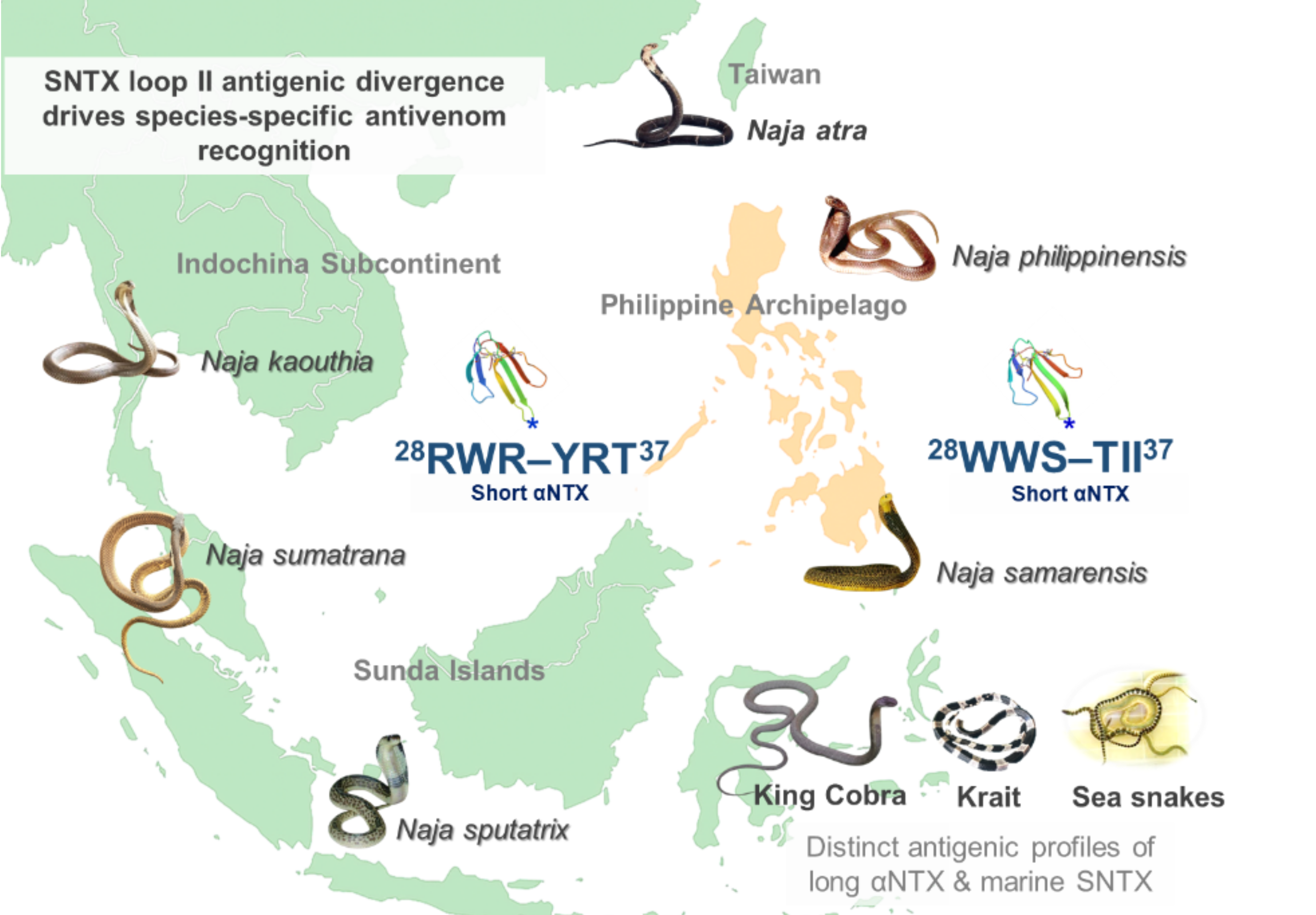

Supplement: S2 File — (PNG) [file pntd.0013859.s002.png]
